# Morphia Injections

**Published:** 1889-11-09

**Authors:** 


### MORPHIA INJECTIONS.

The failure of injections of morphia to induce sleep, although they have completely removed the pain which had hitherto precluded it, is a phenomenon familiar to every practitioner; and akin to this is the common remark of patients that "laudanum only keeps them awake." M. Huchard points to the explanation of this supposed idiosyncrasy in the fact that morphia, unlike chloroform, etc., while depressing functions of the sensorium, tends rather to exalt the excitability of other centres, especially the sensitiveness to slight auditory impressions. Hence, he insists on the necessity of prescribing absolute quiet, and, when practicable, exclusion of light also, in the chamber after an injection of morphia has been administered.

